# Four Years in, What Are the Research Priorities for Long COVID? A Research Priority‐Setting Partnership Between People With Lived Experience, Carers, Clinicians and Researchers

**DOI:** 10.1111/hex.70072

**Published:** 2024-10-24

**Authors:** Aileen Grant, Emma Stage, David Blane, Helen Goss, Jane Ormerod, Stuart McIver, Edward Duncan, Gail Patel, Abi Campbell, Paul Manson, Ganesh Subramanian, Kay Cooper

**Affiliations:** ^1^ School of Health Robert Gordon University Aberdeen UK; ^2^ General Practice & Primary Care, School of Health & Wellbeing University of Glasgow Glasgow UK; ^3^ Long Covid Kids Edinburgh UK; ^4^ Long Covid Scotland Edinburgh UK; ^5^ Nursing Midwifery and Allied Health Professions Research Unit, Faculty of Health Sciences and Sport University of Stirling Stirling UK; ^6^ Perth & Kinross long COVID Support NHS Tayside Perth UK; ^7^ NHS Lanarkshire Blantyre Health Centre Blantyre UK; ^8^ Health Services Research Unit, Institute of Applied Health Sciences University of Aberdeen Foresterhill Aberdeen UK; ^9^ NHS Orkney Balfour Hospital Kirkwall UK

**Keywords:** Long COVID, long‐term sequalae of COVID‐19, postacute sequalae of COVID‐19, postCOVID sequalae, postacute COVID‐19 syndrome, research priorities, research priority setting

## Abstract

**Introduction:**

Long COVID is a life‐limiting condition that affects 65 million people worldwide. It devastates lives with uncertain illness trajectories, and yet, there are many research uncertainties as there is a lack of understanding of its causes, effective treatments and management plans. We set out to identify current research priorities for people with Long COVID, carers, healthcare professionals and researchers.

**Methods:**

A systematic literature review and previous Long COVID priority‐setting exercises identified three broad under‐researched areas of Long COVID research within the fields of Public Health and Health Services Research: symptoms; managing day‐to‐day life; and the emotional impact of Long COVID. We disseminated an elicitation survey that asked for research questions in these areas; responses were analysed and summarised into 42 research questions. A survey was then disseminated, asking respondents to prioritise these 42 questions. Workshops were held with people with Long COVID, carers, healthcare professionals and researchers to analyse responses and agree the top 10 priorities.

**Results:**

The top priorities in order were pharmacological treatment of Long COVID; understanding the pathophysiology; nonpharmacological symptom management; improving public and professional understanding of Long COVID; understanding of the long‐term risks of Long COVID; improving financial and social supports; improving understanding of postviral syndromes; diagnostics; service redesign/pathways; and the well‐being of children with Long COVID.

**Conclusion:**

Four years into the pandemic, there is an emphasis on the need for research on treatment, understanding and support for people living with Long COVID.

**Patient and Public Contribution:**

People with Long COVID and carers were involved in the study design, survey design, dissemination, data analysis, interpretation and reviewing and editing the manuscript.

## 1 Introduction

It is over 4 years since the start of the COVID‐19 pandemic and approximately 2 million people in the United Kingdom and 65 million worldwide self‐report Long COVID, an illness arising from widespread infection of COVID‐19 [1, 2]. These illness estimates are likely to be an under‐representation due to heterogeneity of prevalence estimates and issues with diagnosis [2, 3]. Furthermore, Long COVID has a dynamic population with reinfection of COVID‐19, increasing the risk of developing Long COVID [4]. There are over 200 symptoms associated with Long COVID, across all organ systems in the body, frequently characterised by relapsing and remitting symptoms that cannot be explained by another diagnosis [5]. For many, it is a life‐limiting condition, impacting the ability to engage in everyday activities, such as showering, cooking and watching TV [6], and with uncertain illness trajectories [7]. Long COVID has been found to impact on life as much as cancer; yet, there remains a lack of understanding of its causes, effective treatments and management plans [8].

Research into Long COVID to date has provided epidemiological data and an understanding of the clinical signs and phenotypes of Long COVID. However, there are still many uncertainties [9]. Given the size of the Long COVID population and the risk of Long COVID sequalae [4, 10], there is a need for more research that should be defined by those affected to aid effective use of resources. Research prioritisation involves collaborative work between people with lived experience, carers, clinicians and researchers to consider questions of importance and feasibility. Previous efforts to identify Long COVID research priorities have focused on a better understanding of Long COVID [11], development of a core outcome set [12] and hospital survivors of Long COVID [13]. We set out to bring together people living with Long COVID, carers and clinicians in a Long COVID research priority‐setting partnership (PSP) to identify and prioritise the unanswered questions for future research.

## 2 Materials and Methods

**Figure 1:**
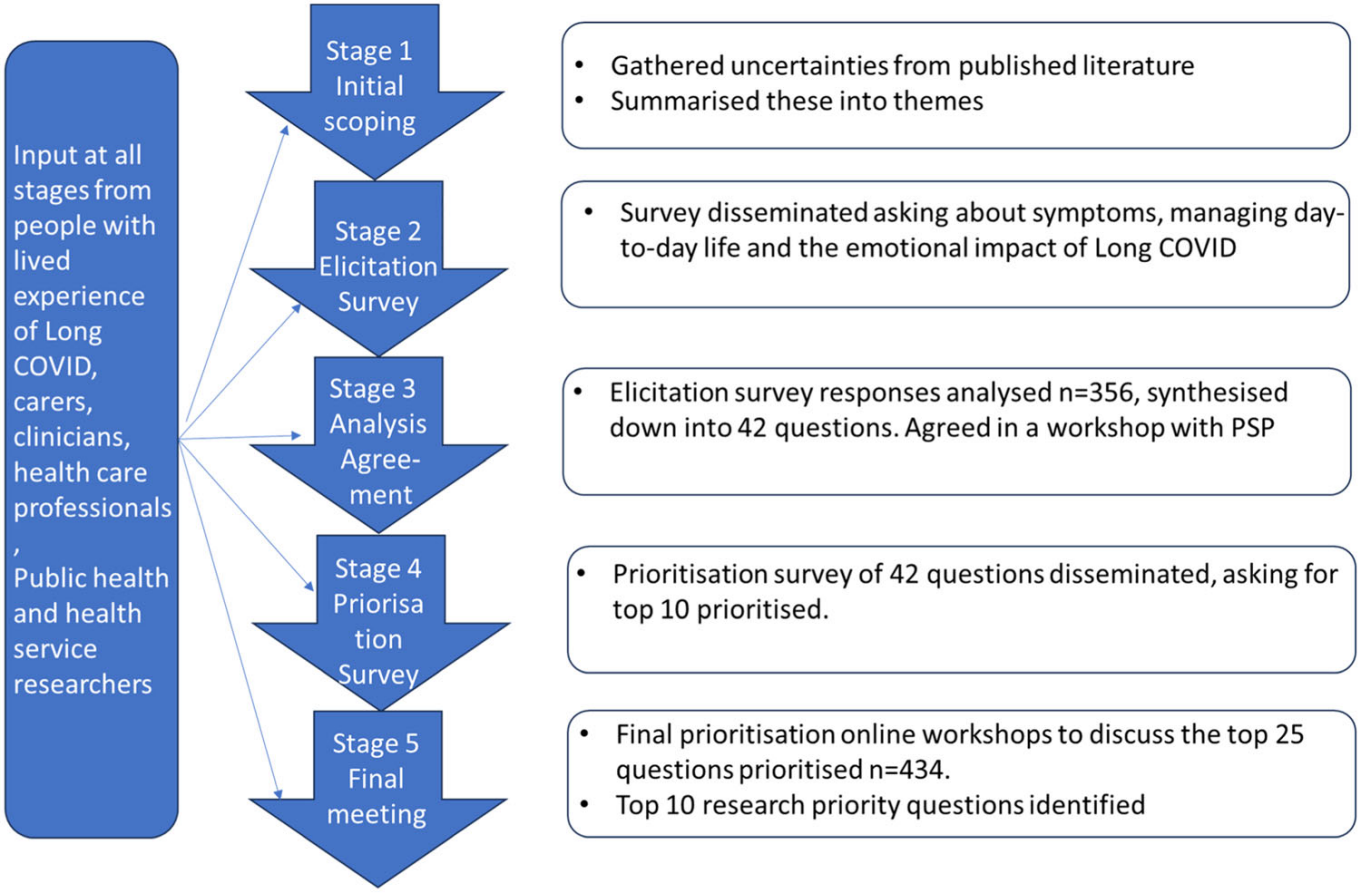
Research priority‐setting process.

Our PSP ran from May 2023 until March 2024 and included the views of adults with self‐reported Long COVID, carers, clinicians and public health and health services researchers. Our PSP participants were invited through organisations such as Long COVID Scotland and NHS Scotland to achieve a diverse sample. Our process is charted in Figure 1.

We searched the literature for evidence uncertainties from published Long COVID systematic reviews and other Long COVID priority‐setting exercises published before May 2023, with the support of an information scientist (PM). We summarised these evidence uncertainties and reviewed them with our PSP into a list of broad research areas within the fields of public health and health services research. We identified three broad under‐researched areas of Long COVID research (symptoms, managing day‐to‐day life and the emotional impact of Long COVID). We developed an elicitation survey that asked people: (1) what questions do you have about Long COVID that you would like to see answered by research within these three areas?; and (2) are there any additional questions that do not fit under these headings? The elicitation survey also contained demographic questions such as age, ethnicity, professional group (healthcare professionals) and symptom duration (people with Long COVID). We piloted this survey with the PSP and made minor changes to enhance readability and ease of completion.

The final version of the elicitation survey was disseminated widely through Long COVID support groups, professional networks and social media accounts of the PSP members (including X and Linkedin) between May and August 2023. We received 356 responses, which were analysed descriptively and summarised into 42 research questions by three members of the research team (AG, ES and KC), before being reviewed and sense‐checked by the PSP.

The 42 questions were then disseminated in a prioritisation survey, using the same methods as those for the elicitation survey, between October and December 2023, where we asked respondents to prioritise their top 10 questions. We worked with an information scientist to develop and run a systematic and comprehensive search of the literature and guidelines to check the top 10 priorities against published research, protocols, and trial registries to identify which questions had been, or were in the process of being, addressed by research. The final top 10 research questions were agreed across two online, video conferencing PSP workshops, each lasting an hour. Ethical approval was not required, but principles of informed consent and safe data handling were strictly adhered to.

## 3 Results

We received 356 responses to the elicitation survey, which generated 42 research questions. Four hundred and thirty‐four responses were received from the prioritisation survey, where respondents prioritised their personal top 10 questions. Most respondents were female, aged between 40 and 60 years and identified as someone with Long COVID (Table 1). Twenty‐five questions were prioritised by at least 20% of the survey respondents (see Appendix A). The PSP identified some overlap between the research questions. During the two online workshops, the PSP conducted an iterative process of reviewing and refining the questions by theme, resulting in these 25 questions being condensed into top 10 priority areas for research for both adults and children (Table 2).

**Table 1:** Participant demographics for elicitation and prioritisation surveys.

	Survey 1 Elicitation *n* = 356 (%)	Survey 2 Prioritisation *n* = 431 (%)
Age		
Under 18 years	3 (0.8)	5 (1.2)
19–39 years	57 (16)	59 (13.6)
40–60 years	230 (65)	291 (67.4)
61–80 years	64 (18)	74 (17)
Did not state	2 (0.6)	2 (0.5)
Gender		
Female	305 (86)	365 (84)
Male	44 (12)	59 (14)
Other	2 (0.5)	4 (0.9)
Prefer not to say	5 (1.4)	3 (0.7)
Ethnicity		
White	337 (94.7)	410 (95)
Nonwhite	15 (4.2)	14 (3.4)
Prefer not to say	4 (1.1)	7 (1.6)
Group		
A person with Long COVID	307 (86)	363 (84)
Parent/carer of child with Long COVID	23 (6.5)	39 (9)
Carer of adult with Long COVID	10 (2.8)	4 (0.9)
Health/social care professional	11 (3)	14 (1.9)
Other	4 (1.1)	11 (2.5)
Did not state	1 (0.3)	0
Geographical location		
Scotland	181 (50.8)	274 (63)
England	142 (39.9)	121 (28)
Northern Ireland	2 (0.6)	2 (0.5)
Wales	8 (2.3)	6 (1.4)
Interactional location	23 (6.5)	28 (6)
In public facing role		
Yes	181 (51)	(n/a)
No	120 (34)	(n/a)
Did not state	55 (15)	(n/a)
Left job/retired because of Long COVID		
Yes	139 (39)	142 (33)
No	111 (32)	165 (38)
Considering	50 (14)	48 (11)
Did not state	56 (16)	76 (18)
Length of time living with Long COVID	*n* = 279 (78%)	*n* = 343 (80%)
Range (months)	3–43	3–48
Mean (SD) (months)	28.5 (11.4)	29.1 (11.5)

**Table 2:** Top 10 prioritised research questions for adults and children.

	Prioritised research question
**#1**	**Pharmacological treatments**
	Are there pharmacological interventions (licensed or unlicensed) that can help with Long COVID symptoms?
**#2**	**Pathophysiology**
	What causes Long COVID?
**#3**	**Nonpharmacological symptom management**
	Are there any nonpharmacological interventions that can reduce symptoms and improve quality of life?
**#4**	**Professional and public understanding**
	How can we improve understanding of Long COVID among health professionals and the general public?
**#5**	**Long‐term risks**
	What are the long‐term health risks of having Long COVID?
**#6**	**Financial and social support/economic impacts**
	How can we improve financial and social support and access to benefits?
**#7**	**Overlap with other postviral syndromes**
	How can we understand postviral syndromes better and their commonalities?
**#8**	**Diagnosis**
	What diagnostics are needed?
**#9**	**Service design/pathways**
	What should Long COVID services and care pathways, for adults and children, look like?
**#10**	**Supporting well‐being in children with Long COVID**
	How can we support children with neurological effects as a result of Long COVID to keep up with their education when they cannot attend school?

For both the elicitation and prioritisation surveys, participants reported similar characteristics: 65% of the elicitation survey and 67% of the prioritisation survey were aged between 40 and 60 years; 95% reported their ethnicity as white; and 86% and 84% identified as a person with Long COVID.

The top priorities in order were for pharmacological treatment of Long COVID; understanding the pathophysiology; nonpharmacological symptom management; improving public and professional understanding of Long COVID; understanding of the long‐term risks of Long COVID; improving financial and social supports; improving understanding of post‐viral syndromes; diagnostics; service redesign/pathways; and the well‐being of children with Long COVID.

## 4 Discussion

Through our co‐designed and conducted PSP, we have identified key priorities for research in adults and children, which span causation, diagnosis, treatment and management, and awareness of Long COVID. There is an emphasis on the need for research on treatment, understanding and support; support is predominantly in health, social care and financial terms but also better appreciation of Long COVID among healthcare practitioners and the public.

Our survey identified 25 research questions, which we synthesised into 10 priority areas. This is unusual in research priority‐setting exercises but, given the overlap between the questions that were prioritised, the PSP felt that this was most appropriate. We have also made the full 25 questions available with the number of responses, so readers are fully informed.

The findings from our PSP are not directly comparable to other Long COVID PSPs, given that each has a unique focus. However, our study is most closely aligned with Houchen‐Wolloff et al., who explored the priorities of hospitalised COVID‐19 patients in 2021 [13]. They identified pathophysiology of Long COVID to be the most pressing priority. Our data were collected 2 years later and there is increasing understanding of the pathophysiology that allows for the development of new treatments; however, it appears that different treatments are required for different clusters of symptoms [14, 15]. It is not surprising that treatments are a top priority, given that the most recent data from the UK Office of National Statistics (ONS) show that 1.1 million, just under 2% of the whole population, are living with Long COVID, 74% said their daily activities were adversely impacted and the majority have been affected for over 2 years [16].

People with lived experience, Long COVID charities, carers, clinicians and healthcare professionals were involved in this process throughout, from conception through to question generation, phrasing and prioritisation. This differs from previous PSP exercises that have focused on core outcome sets [12], hospitalised COVID‐19 patients [13], people with pre‐existing airways disease [17] or that were conducted much earlier in the pandemic [11]. It is not possible to calculate the response rates as the surveys were distributed via social media and professional networks, so the denominator is not known. We do recognise the limitation of our recruitment approach, as Long COVID online support groups are skewed towards those from higher socioeconomic status and those who have sought help [18]. However, our sample included predominantly women with Long COVID, aged between 40 and 60 years, and this demographic is representative of the latest ONS figures on the prevalence of Long COVID in Scotland and England [16]. Our respondents were predominantly people with lived experience and the views of healthcare professionals were not well represented despite targeting professional networks. Response rates to professional surveys have historically been low and are decreasing, leading to unknown levels of bias [19].

Given that the PSP participants were all from Scotland, there may be a bias towards issues more pertinent to Scotland, as there are different approaches to Long COVID management between the four UK nations due to health care being a devolved issue. The most obvious difference is the availability of Long COVID clinics in England but not in Scotland.

## 5 Conclusion

In conclusion, this inclusive and comprehensive PSP conducted between three and 4 years into the COVID‐19 pandemic identifies the most pertinent research questions from the perspective of people with self‐reported lived experience of Long COVID, carers and healthcare professionals. This PSP highlights the most pressing research priorities that need to be addressed, including pharmacological and nonpharmacological interventions, greater awareness of Long COVID, the need for better health and social care and financial support for those affected.

## Author Contributions

**Aileen Grant:** conceptualisation, data curation, formal analysis, funding acquisition, investigation, methodology, project administration, resources, supervision, validation, visualisation and writing–original draft preparation. **Emma Stage:** data curation, formal analysis, funding acquisition, investigation, methodology, resources, software, validation and writing–review and editing. **David Blane:** formal analysis, investigation, methodology, resources and writing–review and editing. **Helen Goss:** formal analysis, investigation, methodology, resources and writing–review and editing. **Jane Ormerod:** formal analysis, investigation, methodology, resources and writing–review and editing. **Stuart McIver:** formal analysis, investigation, methodology, resources and writing–review and editing. **Edward Duncan:** formal analysis, funding acquisition, investigation, methodology and writing–review and editing. **Gail Patel:** formal analysis, investigation, methodology, resources and writing–review and editing. **Abi Campbell:** formal analysis, investigation, methodology, resources and writing–review and editing. **Paul Manson:** methodology, resources, validation and writing–review and editing. **Ganesh Subramanian:** formal analysis and writing–review and editing. **Kay Cooper:** conceptualisation, data curation, formal analysis, funding acquisition, investigation, methodology, project administration, resources, supervision, validation, visualisation and writing–review and editing.

## Ethics Statement

Ethical approval was not required, but principles of informed consent and safe data handling were strictly adhered to.

## CONFLICTS OF INTEREST

The authors declare no conflicts of interest.

## Data Availability

Data are available upon request.

## Appendix A Top 25 Prioritised Research Questions

**Table:**

	Prioritised research question	Respondents
**#1**	**Pharmacological treatments**	**692**
1	Are there any medications (licensed or unlicensed), vaccines, supplements, diets and medicines that can help with Long COVID symptoms? For example, anticoagulants, antiplatelets or ivabradine? Can addressing microclots, hyperactive platelets and endothelitis improve the condition? Can medications used for other autoimmune diseases be effective?	245
5	What medications, supplements and diets are best for managing Long COVID symptoms?	225
17	How do COVID vaccines and antiviral medications affect Long COVID symptoms? Do boosters help?	125
22	Can medications or supplements commonly used for conditions like CFS/ME/MCAS also be tried for Long COVID to see if they offer improvement?	97
**#2**	**Pathophysiology**	**624**
10	Why do people with long COVID experience a wide range of symptoms like fatigue, brain fog, pain and more?	149
11	How does the severity of Long COVID symptoms link to biomarkers, such as microclots and viral persistence?	149
18	Can COVID affect neurological and neurodiverse symptoms and other parts of the body, like menstrual cycles and autoimmune conditions?	124
19	Why do some people get long COVID whereas others do not, and why do symptoms come and go? Can we be sure that these symptoms are caused by COVID, and what is happening in the body to create these symptoms?	123
24	Why do medical tests and X‐rays not show the symptoms of long COVID?	79
**#3**	**Nonpharmacological symptom management**	**536**
4	Can we do anything to make the symptoms of long COVID better or reduce them?	226
8	What advice can be given to people for managing their symptoms? Such as how should we manage mental health issues, joint pain and fatigue? Should we push through exhaustion and dizziness? How do we deal with brain fog in important situations? How much exercise is safe? How do we manage our energy for work and home? How can we avoid a sudden worsening of symptoms? How do we develop better pacing? How do we improve our sleep and manage insomnia? How can we handle chronic pain after COVID?	176
15	How can we improve of quality of life and prevent health from getting worse for those with Long COVID?	134
**#4**	**Professional and public understanding**	**490**
6	How can we improve understanding of Long COVID among the wider population, including managers, coworkers, healthcare professionals, family, friends and society as a whole?	181
9	Why are there no clear public health messages about Long COVID?	166
13	What kind of training can healthcare professionals receive to better identify, treat and refer cases of Long COVID, both in adults and in children?	143
**#5**	**Long‐term risks**	**371**
2	What are the long‐term health risks of having Long COVID, like dementia, cancer or heart problems, and can we lower these risks if someone already has long COVID?	240
16	What happens to people with long COVID over time, can we measure their symptoms and can we tell if their previous health conditions worsened due to COVID?	131
**#6**	**Financial and social support/economic impacts**	**360**
7	When will Long COVID be officially recognised as a disability, and when can people get financial help and access to benefits for it?	177
21	What financial support and benefits are available for those unable to work due to Long COVID, including those who contracted it at work?	97
23	Why is Long COVID not recognised as a work‐related illness?	86
**#7**	**Overlap with other postviral syndromes**	**278**
12	What kind of research can be done to understand postviral syndromes better, building on existing knowledge in these areas?	144
14	Are Long COVID, ME/CFS and fibromyalgia related?	134
**#8**	**Diagnosis**	**229**
3	What tests and scans are needed for Long COVID, and how can we make sure that they are available to patients? For example, testing cortisol levels for conditions like ME/CFS and Long COVID.	229
**#9**	**Service design/Pathways**	**99**
20	What should Long COVID services and care pathways, for adults and children, look like?	99
**#10**	**Long COVID in children**	**75**
25	How can we support children with neurological effects as a result of Long COVID to keep up with their education when they cannot attend school?	75
